# Understanding the Factors Influencing Older Adults’ Decision-Making about Their Use of Over-The-Counter Medications—A Scenario-Based Approach

**DOI:** 10.3390/pharmacy8030175

**Published:** 2020-09-18

**Authors:** Shweta Shah, Aaron M. Gilson, Nora Jacobson, Apoorva Reddy, Jamie A. Stone, Michelle A. Chui

**Affiliations:** 1 Sonderegger Research Center, School of Pharmacy, University of Wisconsin, Madison, WI 53705, USA; srshah6@wisc.edu (S.S.); aaron.gilson@wisc.edu (A.M.G.); areddy7@wisc.edu (A.R.); jamie.stone@wisc.edu (J.A.S.); 2 Institute for Clinical and Translational Research and School of Nursing, University of Wisconsin-Madison, Madison, WI 53705, USA; najacobson@wisc.edu

**Keywords:** medication use, over-the-counter, decision-making, older adults, medication beliefs, knowledge, medication safety

## Abstract

The potential risks of over-the-counter (OTC) medications are often aggravated in vulnerable populations, such as older adults. The elevated patterns of older-adult OTC medication use do not necessarily translate into a greater understanding of these medications or their safety implications. The objective of this study was to assess how older adults’ knowledge, beliefs, and attitudes inform their decision-making regarding OTC use. Situational interviews were conducted in three community pharmacies with 87 older-adult participants to capture how they intended to use an OTC medication. The interviews were transcribed and qualitatively analyzed, generating seven key themes: (1) medication use concerns; (2) following label instructions; (3) wait time until medication effect; (4) responses to medication not working; (5) decision to stop medication; (6) sources of information; and (7) safety implications. This study shows substantial variations in older-adult OTC medication use while providing insight on factors that influence older adults’ appropriate OTC medication use and, in some cases, the potential for harmful effects.

## 1. Introduction

Annual sales data demonstrate that over-the-counter (OTC) medications represent a prominently purchased pharmaceutical treatment class in the United States [1]. Despite the popularity of OTC medications and the ability to obtain them without a healthcare practitioner’s authorization, several safety issues can arise through their use [2]. The safety risks of OTC medications are demonstrated by the significant number of adverse events, hospitalizations, or even deaths that are associated with their use annually [3,4,5,6,7,8]. Such potential risks are aggravated not only by the properties of the OTC medications themselves, but also by the characteristics of the people taking the medications.

Adults aged 65 years or older (older adults) use a disproportionately large number of OTC medications compared to other age groups [9,10], both in terms of overall quantities and rates of use (i.e., daily or weekly). This elevated prevalence is true not only in the U.S., [9] but also internationally [11,12,13,14]. These elevated patterns of OTC medication use do not necessarily translate into older adults having a greater understanding of these medications or of their safety profiles [15,16]. In fact, older adults are at a heightened susceptibility to OTC medication-related harms, including those resulting from interaction with other medications or health conditions [17,18,19], or from generalized side-effects from the OTC medications [20,21]. That is to say, at least for this particular population, even using OTC medications correctly cannot protect someone from problematic outcomes [22].

In addition to the potential harms resulting from interactions with a person’s other medications or health conditions, non-conformity to labelled indications or instructions creates further opportunities for adverse events [23]. The potential for not following label instructions is exacerbated when a person lacks knowledge about the instructions for medication use, including the warning messages accompanying those instructions [24]. Even given the accumulated evidence regarding conditions involved in adverse medication events (e.g., contraindications with other medications, failing to comply with dose recommendations, or not understanding medication instructions), there is a dearth of research documenting the role of patients’ intentions in the occurrence of OTC-related harm.

The current literature does not provide insight into patients’ decisions involving the use of a familiar OTC medication that could either increase or decrease the potential for harm. For example, would a person who is aware of medication instructions, and plans to follow those instructions, consider exceeding the recommended quantity or dosing amounts in the face of slower-than-expected symptom reduction? In addition, does a heightened concern about safety play a protective role against failing to comply with instructions? Alternatively, when a patient’s symptom relief has been less than expected, could a lack of consideration about potential medication-related harms stemming from inappropriate use lead that person to exceed the medication use instructions? These and other considerations lead to the conceptualization and development of a pharmacy-based intervention designed to better understand and address OTC safety issues for older adults.

### 1.1. The Senior Section^TM^

In a systematic effort to decrease older adults’ selection and use of high-risk OTC medications, participatory design [25] and human factors engineering [26] frameworks guided a redesign of community pharmacies’ typical structural layout (called the Senior Section^TM^). In the U.S., medications are classified as either prescription or OTC, with separate legal standards for their accessibility [27]. Many other countries (e.g., Ireland, New Zealand, and Australia) have a classification of “pharmacy only medicines [28,29].” Such medications can only be sold by pharmacists, require pharmacist counseling to relay information to the patient, and are stored in a pharmacy area to which the public does not have access [28]. Conversely, in the U.S., OTC medicines can be purchased off the shelf in stores without pharmacist oversight or consultation to discuss risks. As a result, a principal objective of this intervention was to enhance patients’ awareness of OTC risks and promote opportunities for effective pharmacist–patient communication. A key feature includes a curated inventory of OTCs.

The Senior Section’s OTC medication inventory consists of products that are carefully selected using the Beers Criteria [30]—an evidence-based clinical practice guideline used as a source of older-adult OTC safety information—that have lower risk profiles for the treatment of allergies, coughs/colds, sleep, or pain [31]. As a component of a multifaceted pilot project examining the implementation of the Senior Section and its effectiveness at reducing unsafe medication use, pharmacy customers participated in a scenario-based semi-structured interview regarding their planned use of OTC medications [32]. This data collection process allowed for the evaluation of patients’ feedback about the way that they would use an OTC product that they selected, as well as the factors that they considered could lead either to safe or potentially harmful use. As a result, these data provide an opportunity to examine the impact of patients’ potential OTC medication use on the possibility of unsafe consequences.

Although this analysis is specific to patients’ medication use information, the overall study examining Senior Section implementation has so far generated multiple publications describing different aspects and effects of the intervention. These publications have covered such distinct methods or topics as the explanation of the participatory design influence [31], description of the study protocol [32], descriptive analysis of patient/pharmacist encounters defined through a study data collection form, mixed-methods analysis of patient/pharmacist encounters [33], and qualitative assessment of pharmacy staff reactions to the Senior Section [34]. In addition to these publications, manuscripts currently are being prepared to address the following topics:

- a methods paper describing the task simulation and situational interviewing approach used to capture participants’ decisions when selecting and using OTC medications;
- a methods paper describing the process of classifying types of misuse and operationalizing these misuse constructs;
- a quantitative analysis of the Senior Section’s effect on the occurrence of standardized medication misuse classifications [35];
- a qualitative analysis classifying types of OTC selection processes for older adults, including the results from a latent semantic analysis of older adults’ interviews;
- an industrial engineering analytic method to conceptually diagram older adults’ cognitive decision-making process while considering and selecting an OTC medication.

As a result, this manuscript represents a partial but unique component of the full study, and is meant to address a topic that has received little empirical attention.

### 1.2. Objective

The objective of this study was to assess how older adults’ knowledge, beliefs, and attitudes inform their decision-making regarding OTC use. Specifically, this research identified medication use concerns; the rates of following label instructions; what they consider when deciding to start, continue, or stop medication use; and their preferred resources when seeking more information about taking OTC medications. Finally, these interviews solicited comprehensive information about patients’ considerations of medication safety issues related to their current health condition, potential interactions with current medications, and potential OTC side effects.

## 2. Materials and Methods

A formal qualitative approach [36] was used to explore the process by which older adults would use the OTC medications that they selected to treat a particular symptom profile. Standards for Reporting Qualitative Research [37] were used during the execution of this project and in the preparation of this manuscript. This study is a component of a larger research project that was approved by the University of Wisconsin Institutional Review Board.

### 2.1. Setting

For this pilot study, 3 pharmacies in a Midwestern state were selected from within a single pharmacy chain. The pharmacies were located in communities with populations ranging from about 37,000 to over 200,000 people, representing diverse age, gender, and race demographics [38].

### 2.2. Recruitment

Pharmacy customers were invited to participate in the study via a letter from the pharmacy manager at each site explaining that the store was collaborating with a university research team to learn more about how older adults select and use OTC medications. Eligible participants were 65 years or older and had purchased or considered purchasing an OTC medication in the past 6 months to treat either pain, an insomnia/sleep problem, a cough/cold, or allergies. A cumulative total of 1800 letters were mailed to eligible participants. Study fliers also were placed near the pick-up window at each pharmacy. Both letters and fliers contained instructions for potential participants to contact the study team to learn more about the study

If the older adult preferred, a research team member could initiate a call with the interested customer to explain the study requirements. When a customer’s participation was secured, study materials were mailed, and a time was scheduled to complete an interview at the pharmacy location where they were a regular customer. Participants were paid $20 for completing the interview.

### 2.3. Interview Data Collection

A scenario-based data collection approach was used to explore older adults’ decision-making related to the selection and use of the OTC product. The scenario-based approach had two phases:

1. A simulated task exercise in which the older adults were asked to select an OTC medication by navigating to the required aisle in the pharmacy—the goal of simulation task exercise was to understand how older adults select a medication as they moved through the store;
2. A situational interview, conducted in a semi-private area in the pharmacy, in which the older adults were asked to provide information on how they would use the selected medication—the goal of the situational interview was to capture how older adults intend to use the medication they selected during the simulated task exercise.

This paper focuses on the OTC medication “use” part of the interview that was captured via situational interviewing. A set of structured interview questions was presented to the older adults within a hypothetical situation. Situational interviewing is a scenario-based technique where participants are presented hypothetical situations with the aim of predicting an individual’s performance and behavioral intentions [39]. Situational interviewing is based on the premise that intentions predict behavior, where an individual is asked to prospectively indicate how they would behave in a given situation [40,41]. Situational interviewing has been mainly used for job performance analysis [41,42], and to the authors’ knowledge this is the first time that this technique has been used in health service research.

Each older adult first met a researcher at the pharmacy entrance. The older adult was then asked to pick from one of three conditions (cough/cold/allergy, pain, or sleeplessness) that resonated most with them or that related to the condition for which they usually purchase an OTC. Once the older adult chose a particular condition, the researcher narrated a hypothetical scenario corresponding to that condition. (see Table 1). Each scenario was carefully constructed and vetted by members of the research team, as well as by two practicing pharmacists, to ensure that they were both realistic and relevant scenarios that were appropriate to be treated with an OTC medication.

Once an OTC medication pertaining to their condition was selected, the researcher introduced the “use” section by repeating the scenario to set the stage for the situational interview. The interview guide was constructed with 2 goals in mind: (1) ensure the scenario is relevant to the older adult; (2) narrow the focus of the scenario to keep the older adult on track. The interview guide aimed to elicit responses from older adults about their potential medication use, with questions pertaining to the dosage, frequency of use, and safety concerns about the selected medication. The older adults were allowed to access the label information on the medication box when responding to interview questions. Probes were used to gauge older adults’ rationales and sources of motivation for their potential OTC medication use. Interviews lasted between 10 and 25 min and were audio recorded.

### 2.4. Data Management and Analysis

All the audio recordings were transcribed, and both recordings and transcriptions, once de-identified, were stored in NVivo v.12 for coding purposes. Questions from the interview guide were used to derive the initial coding scheme. Three researchers (AG, AR, SS) independently reviewed two randomly selected transcripts to validate the initial coding scheme and generate additional codes if necessary. The final coding dictionary consisted of 7 main themes and 16 sub-nodes across many of those themes, totaling 23 nodes. Once the coding scheme was finalized, the same three researchers performed open coding on one batch of transcripts each week and met as a group to review, discuss, and reach consensus on coding. Coding was performed and completed during the September to November period, 2019. A master file containing the reviewed and coded transcriptions, along with the final codes, was updated and maintained in NVivo v.12 by one of the three principal analysts (SS).

## 3. Results

A total of 87 older adults took part in the interview across three pharmacy settings, with 29 from each setting. The sample was older adults in the age range of 65–88 years, with the majority of the sample female (66%) and white (96%) and more than half being college graduates (59%). These older adults were on several prescription and/or other OTC medications (in the range of 1–33).

The seven key themes identified for this study, as well as the interview questions generating each theme and its meaning, are listed in Table 2. Exemplar quotations obtained through patient interviews are provided to offer qualitative support for each of the key domains. When necessary, patient quotations were edited to remove non-substantive interjections or partial phrases that were eventually stated in their entirety. The frequencies presented below represent descriptions of the sample’s responses (from 87 older adults) to a set of structured stimuli (interview questions), and are not meant to convey a sense of generalizability to a wider population. These frequencies are merely meant to display the degree of consensus within the sample around particular thematic issues.

### 3.1. Medication Use Concerns

Overall, 20 older adults expressed certain apprehensions related to their selected OTC medication, which influenced their decisions about the dosage, frequency, and timing of their medication use. These concerns were categorized as older adults’ (1) activity concerns, (2) medication interaction concerns, and (3) health concerns.

#### 3.1.1. Activity Concerns

Of the older adults who stated having medication use concerns, about a third related to concerns involving how their OTC medication use interacted with their routines and/or daily activities.

*“And if I do [an activity] to stress, then, I can’t take it [medication] at night, though. It messes with my sleep.”*

#### 3.1.2. Medication Concerns

A section of older adults with medication use concerns indicated that they modified their OTC use based on when they take their other medications.

*“About an hour after breakfast or so… I never take it together with my Synthroid. I just don’t feel like it’s a good idea.”*

#### 3.1.3. Health Concerns

Finally, about a half of the older adults based their potential use of the selected OTC medication on experiences with past or current health issues.

*“And you know, my symptoms are most in the morning, and then towards afternoon and that, well, they get better. And then towards the evening, they deteriorate because, you know, when you have asthma, you always deteriorate in the evening, not terribly, but you do. So anyway, I would take it in the morning, and I’d look out for, I don’t go right outside. I keep the air conditioning going, and I’m very careful because, you know, I really do have severe asthma.”*

### 3.2. Following Label Instructions

Almost half of all older adults stated an intent to follow instructions on the medication bottle when describing their potential OTC medication use. However, the degree of intent seemed to vary between those who indicated following label instructions without deviation and those who were willing to diverge from label instructions based on certain situations.

#### 3.2.1. Following Label Instructions without Deviation

For those older adults who reported a commitment to following label instructions, most emphasized a firm, unwavering intent to use the selected medication based on the box information.

*“I would follow the label to a T because I believe in doing that…..I’m pretty rigid about following what it says on the label because I feel this has been tested, and that’s the information that’s given for your safety and well-being.”*

*“I am very strict at following what it says, because I hate taking medication, and I hate taking too much, and I always worry about side effects or doing something wrong. So I, in fact every day, even when I take it, I look at the container, and I make sure I’m taking the right thing. And I’ll look at it, and like, okay, that’s my allergy pill, and then I’ll take one every morning.”*

#### 3.2.2. Conditional Following of Label Instructions

A small but notable portion of older adults admitted to a level of uncertainty about following label instructions, depending on certain factors such as lack of appropriate knowledge or a concern about drug overdose.

*“Would I exceed the six? Well, there’s a possibility, but I’m not sure that I would.”*

*“Well, I read the label to see how many you should take, you know, and how often you should take it. But very seldom do I take it every four hours or every six [label instructions]. I just take it when I need it, you know. And then I make sure that if I do need it more, if it’s that bad, then I do know when I took it the first time, so I know if it’s within limits. But I’m not a, I don’t like to take a lot of pills if I don’t have to.”*

### 3.3. Wait Time to Medication Effect

Older adult responses varied based on their interpretations of the “amount of time to wait for medication to work.” Some older adults responded with a wait time of only about a few hours before taking the next dose, while most others stated a more prolonged wait time to see if the medication was working—usually a couple of days.

*“I’d probably, I mean, I wouldn’t take anything else until, you know, if this is good for four hours, and it says two soft gels every four hours, so I would probably wait four hours.”*

*“Probably about, well, if I, if my symptoms didn’t change, you know, and you didn’t mention wheezing and gasping and all of that. If they didn’t change, I would wait three days, and I’d see how I’m doing.”*

### 3.4. Responses to OTC Medication Not Working

Almost every older adult mentioned using at least one strategy in response to the possibility that the selected OTC medication had insufficient or no therapeutic effect. These strategies included switching to an alternate medication, taking more of the same medication, switching to a non-pharmacological treatment, considering seeing a healthcare professional, or taking no action (i.e., dealing with it).

#### 3.4.1. Switching Medication

About a third of older adults intended to switch to a different medication (either to a different OTC medication or a prescription product) in response to unsatisfactory therapeutic outcomes. What differed among respondents was the amount of time for which the unrelieved symptoms were endured—while most would wait for at least a couple of days (delayed switch), a few would make the switch after only one dose (immediate switch).

- Delayed switch:
  “I might come back and get something else. Say this just not doing the trick, and then they usually will find something stronger for me.”
- Immediate switch:
  “You know, if it was getting close to bedtime, I’d probably just take a couple more Benadryl and an aspirin, and then just, you know, hope that that would do the trick.”

#### 3.4.2. Taking More of Same Medication

Over half of older adults reported the intent to take more of the same medication. Similar to medication switching, responses were divided depending on immediate reactions after one dose or decisions to take more of the same medication after a couple of days.

- Immediate reactions:
  “If I woke up, and if it was bothering me, yeah, I’d take another dose in four hours.”
- Delayed reactions-:
  (Interviewer: *“And well, so if it didn’t work, what would you do, would take some more pills?”*)
  “Yeah, I’d take a couple more.”

#### 3.4.3. Switching to Non-Pharmacological Treatment

Few older adults preferred switching to non-pharmacological treatments, including herbal, ayurvedic, and/or home remedies, in situations where their selected OTC medication did not alleviate their symptoms.

*“I would not be happy. I would, I’d get some herbal tea, or, you know, drink orange, I mean, drink an orange juice. I’d be taking one of those other ones, the Emergen-C thing. I’d be doing something to help boost the immune system, making sure I ate better, drank more water, something.”*

*“I mean, you know, I possibly may take a warm shower. I generally don’t, the muscle aches and etc. are usually generalized, so using ice isn’t an option because it’s all over.”*

#### 3.4.4. Considering Seeing Healthcare Professional

A vast majority of older adults preferred consulting and/or or visiting a healthcare professional (including, but not limited to, physicians, pharmacists, and nurse practitioners) whenever their selected OTC medication did not produce the desired outcomes.

*“I would probably go back and talk to the pharmacist and say, I’m thinking about getting this arthritis one, unless you have something else I should, you’d recommend, and see what he recommends rather than waste my money on another medication that might not work. I really rely on the pharmacist.”*

*“Well, I would probably, I have [insurance], so they have a, my healthy chart or something. MyChart, yeah, yeah. So I’d go on there, and I’d e-mail my doctor, and then the nurse would get it and, because I get a fast answer doing that.”*

#### 3.4.5. Dealing with the Problem

Around a third of the older adults preferred to take no further pharmacological or non-pharmacological action if the selected OTC medication did not work. Instead, they would just suffer through the symptoms, hoping to feel better soon.

*“Well, like I said, I do a lot of, you know, relaxation. If nothing really works and then I determine, and this would be completely different if I actually had a job where I had to be somewhere, but if I really couldn’t fall asleep, and I tried everything, again, I would get up and read or watch TV. I wouldn’t try and take something that wasn’t working. I might even, I have already done yoga. You know, just let my husband sleep, and I run into the other room. And, but that’s what I would do.”*

*“I think I’d probably let, I’ve been a distance runner, and so I know you have to suck up some of it, and I err more on the side of letting something go.”*

### 3.5. Decision to Stop Medication

Overall, a large proportion of older adults provided explicit explanations about how they would decide to stop taking the medication, with about two-thirds indicating that they would cease the medication use upon symptom relief.

*“I’d only take it until it, the pain started going away. There’s no need to take it, you know, if you don’t have the pain.”*

*“If it keeps working. If I get a lot, enough congestion up that I feel I don’t, I’m not doing too badly, then I just stop taking it for a while, and then I have some when I need it again.”*

### 3.6. Sources of Information

All the older adults identified the primary source that they would consult if they needed information about their selected OTC medication. Such sources comprised pharmacists, other healthcare professionals (such as physicians or nurses), or other resources not directly involving healthcare professionals. A notable minority of older adults stated that they would regularly use more than one source when they sought OTC-related information.

#### 3.6.1. Pharmacists

More than half of the older adults stated that they would attempt to obtain help from pharmacists by either calling them or visiting the pharmacy to obtain information about their selected OTC medication.

*“I would see the pharmacist. And we love the pharmacists here at [the pharmacy]. They’re really informative.”*

Amongst these older adults, a majority mentioned a clear preference for first seeking information from the pharmacists or going to the pharmacy before approaching other sources.

*“I think I would go to the pharmacist first, and, or maybe check it out online. See if there is something like that because there’s some, a few sources that are reputable. I guess WebMD is kind of okay.”*

#### 3.6.2. Other Healthcare Practitioners

Another quarter of older adults stated that they would obtain information about their selected OTC medication from other healthcare professionals.

*“We’re on the MyChart with our physician… And so if I had any questions, I would probably send a MyChart message to my doctor.”*

For those older adults who sought information from healthcare practitioners, 43% mentioned that they would initially try to obtain further information from the other healthcare providers before (if at all) seeking information from other sources.

*“Probably go to the doctor first and see what he said.”*

#### 3.6.3. Other Sources

There were some older adults who identified relatives, friends, and other non-practitioner sources as their main information source.

*“…just talk to friends. I’m not a big doctor person.”*

As with the other categories within this thematic domain, there was a small proportion of older adults (38%) who preferred to access non-professional sources as a first attempt to gather information, before relying on healthcare sources.

*“Epocrates. And it’s the same drug program that my doctor uses. So I know we’re on the same page with that one, so… I would Google the medication. And well then, I would call the pharmacist and say, okay, what do you think of this? You know, if I couldn’t find enough information on the drug itself, I would call the pharmacist before I call my doctor”.*

### 3.7. Safety Implications

Older adults’ perceptions of the safety of their selected OTC medication were assessed by exploring their knowledge and beliefs about OTC-induced health problems and interactions with their current health conditions and with the other medications that they take. Older adults expressed different degrees of confidence about the safety of the OTC medication that they selected. Responses to safety implications were categorized either as detailed responses supported with rationale and motivations (elaborative responses), firm unelaborated responses represented by yes/no responses (declarative responses), and those indicating a lower degree of certainty containing some equivocal statement (e.g., “Yes. I guess so”) (equivocal response).

#### 3.7.1. OTC-Induced Health Problems

Almost every older adult responded when asked whether they knew of any side-effects or health consequences from taking their selected OTC medication, with 10% providing a declarative response and 14% providing an equivocal response. However, three quarters of the older adults chose to support their response by sharing their past experiences, or providing a rationale or motivation.

*“Yes, it can cause ulcers, the doctor said. That’s why they don’t, well, that’s why my doctor didn’t want me to really take a lot of ibuprofen, where he was going to the acetaminophen area. And even now he knows I take it, but he’s fine with, you know, just limited dose on it. But that’s what he said. It could lead to stomach ulcers”.*

*“No. I mean, you know, when you read the back it always says if you have high blood pressure, if you have diabetes, if you have this or that or whatever, don’t do this, and I don’t fall into any of those categories. You know, I have a little bit of aches and pains from arthritis. The medications I take are pretty stable. I’ve been on them for a long time, so.”*

#### 3.7.2. OTC Interactions with Other Medications

All the older adults provided feedback about the potential for OTC interactions with their other medications, with a quarter of older adults stating a declarative response and a few stating an equivocal response. About two thirds of the older adults elaborated their responses.

*“With what I currently take, yes. And I do have a list of medications because sometimes I take, I have to take an opioid, and I have two different opioids I take. And I have a list as to whether I can take Tylenol, yes or no with each of the opioids, and Aleve, yes or no with each of the opioids, because I can’t remember it. So I write it down. And if I were unsure, if I couldn’t find my list, then I would call over here and say, can I take Aleve and Tramadol at the same time? That’s where I got this information initially from.”*

*“No. I don’t, if I have to take a hydrocodone pain pill for my back, I never take [the selected medication] along with it, never, ever, because they’ve both got acetaminophen in them, and I’m conscious of that”.*

#### 3.7.3. OTC Interactions with Health Conditions

All the older adults understood the potential for interactions between the selected OTC medication and their current health conditions, with about a third offering a declarative response and few offering an equivocal response. Another almost half provided details about their past experiences to support their responses.

*“The gastric reflux, which is not really much of an issue right now. But if I were having problems with that, I’d have to switch over to the acetaminophen, even though it’s less effective.”*

*“Pretty much. It just says heart attack and stroke. Allergy, I’ve never had an allergy. Stomach bleeding, I don’t have ulcers or bleeding products. I don’t take a blood thinner, so I’m not at high risk for that, these issues.”*

## 4. Discussion

These findings provide insights into the gauging factors that influence older adults’ appropriate OTC medication use and, in some cases, the potential for harmful effects. Older-adult use of OTCs varies widely and is affected by such factors as personal beliefs, [43] acquired knowledge from various sources, and previous experiences with OTC medications. Most study older adults properly indicated that the primary factor influencing their decision to stop taking the selected medication was the alleviation of symptoms. That is, most older adults reported that they would not become accustomed to taking the medication in the absence of symptomology that warrants such use. This complies with the American Geriatric Society’s recommendation to limit the use of non-steroidal anti-inflammatory drugs (as mostly selected by older adults in our study) to the shortest duration [44].

Medication use concerns were uncommon, but the concerns that were stated related to the possibility of interactions with other medications and/or already-existing health conditions. Being cautious of drug–drug interactions is essential, considering that the concurrent use of OTC medications with other OTCs or prescription drugs has long been one of the primary causes of medication mismanagement in older adults, often leading to an adverse drug event [45].

The findings also suggested that, for many people, a preference for a specific OTC medication, including comfort with its features (e.g., pill size, dissolvability) and past effectiveness, can increase the likelihood that that medication is selected to treat symptoms for which the medication may not be indicated. As a result, decisions to take a medication in the presence of broad and diverse symptomology have the potential to lead to less rigorous considerations of its safety profiles, which creates enhanced opportunities for inappropriate use and adverse events.

A notable portion of the participating older adults in our study was highly motivated to follow label instructions while taking their selected medications and claimed to not deviate from the label instructions at any times, while a few expressed that their likelihood of complying with the label was dependent on various situations. In comparison, the rates of following instructions for prescription medications for chronic conditions have been 50% and have remained unchanged for many years [46,47,48]. The findings from this study should be replicated with a larger sample, since taking more medication than the instructions indicate poses a greater risk to older adults, resulting in unsatisfactory therapeutic results and consequent adverse health outcomes [49].

In the face of either insufficient immediate or gradual symptom relief, almost two thirds of the older adults would wait an appropriate amount of time before considering alternative treatment strategies. It is important that, of all the alternative strategies considered (e.g., taking more of the same medication, switching medications, or using non-pharmacological treatments), more patients expressed a willingness to consult with a healthcare professional when a medication did not work to their satisfaction. This approach is especially critical when the older adult was also considering whether to take more of the same medication or switch to another medication under Beer’s criteria (e.g., ibuprofen—a popular selection by older adults in the study), which carries higher risks for older adults [30].

Since deficits in patient knowledge about their OTC medications may lead to adverse outcomes [50], a promising finding was that patients’ most common formal source of information when they had questions about their selected medications was pharmacists. Obtaining information from reliable professional sources, such as pharmacists or other healthcare professionals, is critical to promoting safer decision-making regarding OTC medication selection and use [51].

Most, if not all, of the older adults who participated in this study held clear ideas about the safety implications of their selected medications and could offer explicit reasons for their safety beliefs. However, this study was not designed to determine whether these explanations or beliefs were valid. As a result, pharmacists, being the first line of contact for patients regarding medication-related issues, must seize every opportunity to engage and counsel patients about the safety implications of OTC medications [2,52].

### Limitations

Although encouraging, these study findings should be viewed in light of several design limitations. First, the hypothetical nature of the scenarios required older adults to imagine themselves having particular symptoms and to report on their behavior as if they were currently experiencing those symptoms. We attempted to mitigate this issue by allowing the older adults to select a scenario that they had experience with. It is possible though that the self-reported potential behaviors may not translate into the same actions under real-world circumstances in which the person is actively experiencing symptoms. Second, and similarly, responses to questions aiming at gauging older adults’ knowledge (e.g., “What health problems might come up from taking this medication?”) may not always translate into action. Ideally, elaborate knowledge of the safety profile of the selected OTC medication including contraindications and side effects would translate into timely and safe medication use. However, responses about knowledge may not be indicative of behavior. As indicated above, many older adults reporting declarative perceptions about OTC medication safety, whether related to the development of health problems from such use or OTC medication interactions with other drugs or health conditions, does not necessarily guarantee that those perceptions are valid, nor does it ultimately translate into the safe use of that medication. Third, although older adults’ interview responses targeted a particular class of selected medications (either pain, sleep, or cough/cold/allergy), it is not possible to generalize those responses across all health conditions for which older adults purchase and consume OTC medications. For example, an older adult who claims to strictly follow label instructions for an allergy medication might not report the same level of compliance with the label of his or her sleep medications. Fourth, it is possible that older adults responded to some questions in a manner that would be viewed favorably by others, which could represent a social desirability bias. As an example, older adults’ claims of following their medication label instructions and/or consulting a healthcare professional could reflect more their belief about what they “should” be doing (ideally) instead of what they would do actually. It should be noted that these results only represent older adults who volunteered to participate in the study, and may differ from those who refused to participate in the study. For example, the high reported rates of following medication instructions without deviation could be due to a social desirability bias and might differ from those who refused to participate. Given that the researchers relied on older adult voluntary participation and self-reports of their activities related to the hypothetical use of an OTC medication, the potential influence of social desirability bias cannot be ruled out. Finally, interviewers’ probing techniques seemed to occasionally lead to an interruption of older adults’ natural decision-making process on how they would use the selected OTC medication and may have unintentionally elicited different responses. A prominent example of this was found for interviewers’ probes relating to older adults’ wait times to determine whether the selected OTC medication was working—an interviewer asked for the general amount of wait time (i.e., leaving it to the older adult to interpret the relevant timeframe), while another explicitly asked for the number of days and/or hours. This inconsistency may have created different contexts within which patients responded to a particular interview question.

## 5. Conclusions

Older adults’ decision-making for OTC medication use varied substantially, and was influenced by their knowledge, perceptions, and beliefs about their selected OTC product or even medications in general. Among the many encouraging findings related to this sample of older adults, such as intention to follow to label instructions, medication safety concerns, and avoiding medication overuse, the reliance on health care professionals and other resources to obtain information on OTC medications was noteworthy. Especially notable was the prominent reliance on pharmacists as a key resource for navigating the intricacies and implications of using OTC medications, including switching to other medications when necessary. Cumulatively, these results suggest that many older-adult behaviors have a high potential to contribute to safer medication use.

## Figures and Tables

**Table 1:** Sleep, pain, and cough/cold/allergy scenarios.

Sleep scenario	Recently, you have been having (more) difficulty falling asleep or staying asleep. You are here at Shopko to look for a medication that can help you sleep.
Pain scenario	You are having soreness and muscle aches. It is not bad enough to call your doctor. You have not taken any medication to help with these aches yet. You are here at Shopko to look for a medication that can help you feel better.
Cough/cold/allergy scenario	For the past three days you have had a runny nose, sore throat, felt “stuffy”, and your head is congested. You do not have a fever and it is not bad enough to call your doctor. You have not taken any medication for your symptoms yet, but you are here at Shopko to look for a medication that can help you feel better.

**Table 2:** Definitions and origins of the principal themes in the study.

Theme	Definition of Theme	Interview Question Generating the Theme
Medication use concerns	Older-adult concerns potentially influencing their decisions about the dosage, frequency, and timing of the selected OTC medication.	How would you take this medication? - How often? - How many? - What time of day?
Following label instructions	Older-adult intentions to follow label instructions on the medication bottle when describing their potential OTC medication use.	How would you take this medication? - How often? - How many? - What time of day?
Wait time to medication effect	Older-adult interpretations of the amount of time they would wait for the medication to begin alleviating symptoms of a condition.	How long would you wait to see if the medication is working?
Response to medication not working	Older-adult perceptions of strategies in response to insufficient or no therapeutic effect of selected OTC medication.	What would you do if the medication did not work? - Would you take another pill that night? - Increase the dose the next day?
Decision to stop medication	Older-adult explanations about how they decide to stop taking a medication.	How would you decide when to stop taking the medication? - How long would you keep taking it if it was working?
Sources to seek information	Older-adult preferences of reliable sources to consult in case they require information on how to use the selected medication.	What would you do if you needed more information about how to take this medication?
Safety implication	Older-adult knowledge and beliefs on problems arising from the selected medication and interactions with existing health conditions/other medications.	What health problems might come from taking this medication? Is this medication safe to take with the other medications you take? Is this medication safe to take with your health conditions?
